# Unveiling the inhibition mechanism of *Clostridioides difficile* by *Bifidobacterium longum via* multiomics approach

**DOI:** 10.3389/fmicb.2023.1293149

**Published:** 2023-11-08

**Authors:** Sung-Hyun Jo, Hyo-Jin Jeon, Won-Suk Song, Jae-Seung Lee, Ji-Eun Kwon, Ji-Hyeon Park, Ye-Rim Kim, Min-Gyu Kim, Ji-Hyun Baek, Seo-Young Kwon, Jae-Seok Kim, Yung-Hun Yang, Yun-Gon Kim

**Affiliations:** ^1^Department of Chemical Engineering, Soongsil University, Seoul, Republic of Korea; ^2^Department of Laboratory Medicine, Kangdong Sacred Heart Hospital, Hallym University College of Medicine, Seoul, Republic of Korea; ^3^Department of Biological Engineering, College of Engineering, Konkuk University, Seoul, Republic of Korea

**Keywords:** *Bifidobacterium longum*, *Clostridioides difficile*, microbe-microbe interaction, molecular mechanism, multiomics

## Abstract

Antibiotic-induced gut microbiota disruption constitutes a major risk factor for *Clostridioides difficile* infection (CDI). Further, antibiotic therapy, which is the standard treatment option for CDI, exacerbates gut microbiota imbalance, thereby causing high recurrent CDI incidence. Consequently, probiotic-based CDI treatment has emerged as a long-term management and preventive option. However, the mechanisms underlying the therapeutic effects of probiotics for CDI remain uninvestigated, thereby creating a knowledge gap that needs to be addressed. To fill this gap, we used a multiomics approach to holistically investigate the mechanisms underlying the therapeutic effects of probiotics for CDI at a molecular level. We first screened *Bifidobacterium longum* owing to its inhibitory effect on *C. difficile* growth, then observed the physiological changes associated with the inhibition of *C. difficile* growth and toxin production *via* a multiomics approach. Regarding the mechanism underlying *C. difficile* growth inhibition, we detected a decrease in intracellular adenosine triphosphate (ATP) synthesis due to *B. longum*–produced lactate and a subsequent decrease in (deoxy)ribonucleoside triphosphate synthesis. *Via* the differential regulation of proteins involved in translation and protein quality control, we identified *B. longum*–induced proteinaceous stress. Finally, we found that *B. longum* suppressed the toxin production of *C. difficile* by replenishing proline consumed by it. Overall, the findings of the present study expand our understanding of the mechanisms by which probiotics inhibit *C. difficile* growth and contribute to the development of live biotherapeutic products based on molecular mechanisms for treating CDI.

## 1. Introduction

The human gut is a habitat for diverse and numerous microorganisms (∼10^13^ to 10^14^). However, an imbalance in the gut microbiota, also known as dysbiosis, can lead to infections caused by various opportunistic pathogens, which can further lead to various diseases as well as severely affect the immune system of the host (Pickard et al., 2017). *Clostridioides difficile*, a representative enteric pathobiont, is a gram-positive anaerobe that can cause disorders ranging in severity from mild diarrhea to acute colitis and death (Leffler and Lamont, 2015). *C. difficile* infection (CDI) constitutes a leading cause of nosocomial disease and is responsible for ∼30% of all antibiotic-associated diarrhea incidence, reportedly causing a total of 462,100 cases and > 13,000 deaths in the United States in 2017 alone (McFarland et al., 2016; Guh et al., 2020). In general, antibiotic administration constitutes the main risk factor for CDI as it causes dysbiosis and leads to the loss of colonization resistance against *C. difficile* (Deshpande et al., 2013; Schubert et al., 2015). However, as a standard treatment option for CDI, antibiotic therapy, including those of metronidazole, vancomycin, and fidaxomicin, is preferentially used (Kelly et al., 2021). Eventually, antibiotic therapy causes gut microbiota perturbation, resulting in a very high CDI recurrence rate (∼25%), and approximately 35∼65% of patients with recurrent CDI (rCDI) experience multiple rCDI (Crowther and Wilcox, 2015; Collins and Auchtung, 2017).

Therapeutic strategies involving live microorganism intervention can overcome the aforementioned problems with antibiotic therapy. Thus, recently, approaches to leverage probiotics, which are beneficial gut microorganisms, for preventing and treating CDI and rCDI have been proposed (Crow et al., 2015; Valdés-Varela et al., 2018; Pal et al., 2022; Zhang et al., 2022). Although several preclinical studies have reported the potential of probiotics for improving CDI outcomes, evidence regarding the preventive and therapeutic efficacies of probiotics for CDI was insufficient in several meta-analyses of clinical trials (Goldenberg et al., 2017; Heil et al., 2021; Kelly et al., 2021; Pal et al., 2022). Consequently, based on these studies, the American College of Gastroenterology recently recommended that probiotics should not be employed to prevent CDI and rCDI in patients receiving antibiotic therapy (Kelly et al., 2021). This recommendation was based on the inconsistent outcomes of several trials involving probiotics for CDI alongside the poor quality of the evidence regarding their efficacy. This problem stems from a lack of understanding and research regarding the mechanisms by which probiotics improve CDI symptoms, thereby leading to inadequate study designs involving inappropriate strain selection and efficacy assessment methods (Pal et al., 2022).

To date, several studies have reported various mechanisms by which traditional probiotics (*Lactobacillus* spp. and *Bifidobacterium* spp.) inhibit *C. difficile* growth and virulence. First, probiotics inhibit *C. difficile* growth by producing antimicrobial substances such as organic acids, hydrogen peroxide, and bacteriocin. Naaber et al. (2004) compared the antagonistic activity of 50 *Lactobacillus* spp. strains against 23 *C. difficile* strains and observed that *lactobacilli* with *C. difficile* growth-inhibitory activity produced more lactate and hydrogen peroxide. Furthermore, reportedly, *Lactobacillus reuteri* inhibits *C. difficile* growth by producing a bacteriocin known as reuterin (Spinler et al., 2017). Probiotics can also decrease *C. difficile* virulence by reducing the expression or degrading *C. difficile* toxins. Valdés-Varela et al. (2016) compared the protective effect of 20 *Bifidobacterium* spp. and *Lactobacillus* spp. strains against the cytotoxicity of *C. difficile* culture supernatants, confirming that *Bifidobacterium longum* and *Bifidobacterium breve* exhibit antitoxin effects and that these strains effectively reduce the amount of toxin in the supernatant. In addition, probiotics may prevent *C. difficile* colonization by inhibiting *C. difficile* adhesion to intestinal epithelial cells. Reportedly, *L. acidophilus La-5* culture supernatants reduce *C. difficile* adhesion to the human intestinal epithelial cell lines, Caco-2 and HT-29, further alleviating the cytotoxicity of *C. difficile* supernatants (Najarian et al., 2019). Despite numerous efforts to elucidate the mechanism underlying *C. difficile* growth inhibition by probiotics, our understanding regarding this mechanism at the molecular level remains very limited. For example, the molecular-level interactions between probiotics and *C. difficile* and the resulting changes in metabolism associated with the *C. difficile* growth inhibition and virulence factor regulation remain unexplored. Multiomics study, a holistic qualitative and quantitative analysis of biomolecules in a biological system, can help fill the knowledge gaps regarding the mechanism of action of probiotics against CDI (Kim et al., 2020; Song et al., 2021; Lee et al., 2022; Park et al., 2022). Therefore, this study aimed to investigate the interaction and inhibition mechanisms at the molecular level between *Bifidobacterium* strains and *C. difficile* through a multiomics approach.

Herein, we selected *Bifidobacterium* strains that exhibited the highest growth inhibition on anaerobic coculture with *C. difficile* and investigated these inhibitory mechanisms *via* liquid chromatography–tandem mass spectrometry (LC-MS/MS) based multiomics analysis. First, we cocultured five *Bifidobacterium* strains with *C. difficile* and selected *Bifidobacterium* subsp. *longum* (*B. longum*), which showed the highest growth efficiency. Next, we assessed lactic acid and acetate production by *B. longum* and their contribution to its antimicrobial activity against *C. difficile* using spot-on-lawn assay and organic acid quantification. Finally, we analyzed changes in the quantitative proteomic and metabolomic profiles of *C. difficile* upon coculture to elucidate the growth inhibition mechanism of *B. longum* on *C. difficile*. Through this approach, we were able to observe global changes in the proteome and metabolome of *C. difficile* due to *B. longum* and confirm that essential regulated metabolic processes (including lactate reduction *via* the lactate dehydrogenase complex, proline metabolism, butyrate metabolism, translation, and nucleoside phosphorylation) were involved in *C. difficile* growth inhibition and reduced toxin production. We think that the results of this study can not only broaden our understanding regarding the action mechanism of probiotics against *C. difficile* but also contribute to the development of live biotherapeutic products based on molecular mechanisms for CDI treatment in future.

## 2. Materials and methods

### 2.1. Bacterial strains and culture conditions

*Clostridioides difficile* Korean Collection for Type Culture (KCTC) 5009 (i.e., ATCC 9689), *Bifidobacterium longum* subsp. *longum* KCTC 3128 (i.e., ATCC 15707), *Bifidobacterium longum* subsp. *infantis* KCTC 3249 (i.e., ATCC 15697), *Bifidobacterium animalis* subsp. *animalis* KCTC 3219 (i.e., ATCC 25527), *Bifidobacterium animalis* subsp. *lactis* KCTC 5854 (i.e., DSM 10140), and *Bifidobacterium breve* KCTC 3419 (i.e., ATCC 15701) were obtained from the KCTC. All the strains were cultured with reduced Reinforced Clostridium Medium (RCM) in an anaerobic chamber (90% N_2_, 5% CO_2_, and 5% H_2_; Coy Laboratory Products, MI, USA) at 37°C.

### 2.2. Cocultivation of *Bifidobacterium* spp. and *C. difficile* for probiotics screening

*Clostridioides difficile* and five strains of *Bifidobacterium* spp. were cultured alone until midlog phase. Then, the cultures were centrifuged at 13,500 rpm for 3 min, and the cell pellets were resuspended in fresh RCM broth at 1 × 10^8^ colony forming units (CFU)/mL for *Bifidobacterium* spp. and 5 × 10^6^ CFU/mL for *C. difficile*. 12-mm transwells (3401, Corning, NY, USA), round plastic wells with permeable membrane inserts, were used for the cocultivation of *Bifidobacterium* spp. and *C. difficile*. *Bifidobacterium* spp. (0.5 mL) was inoculated in the insert of the transwell, and 1 mL *C. difficile*, which is 1/10 of the inoculated CFU of *Bifidobacterium* spp., was inoculated in the bottom chamber. The bacterial cells were cocultured for 10 h. A *C. difficile* monoculture control (grown alone) had 0.5 mL fresh RCM in the insert. For the growth assay, the OD_600_ of the bottom chamber after 10 h coincubation was measured using an UV spectrophotometer (Multiskan Go, Thermo Fisher Scientific, MA, USA). The pH of the culture supernatant was measured using a pH meter (815600, Thermo Fisher Scientific, MA, USA).

### 2.3. Antimicrobial activity assay

To determine the antibacterial activity of *B. longum* KCTC 3128 against *C. difficile*, a spot-on-lawn assay was performed. *C. difficile* (5 × 10^6^ CFU/mL) was spread on RCM agar plates using a sterile swab. Then, an inoculum was prepared using a 10-fold serial dilution of 5 × 10^9^ CFU/mL *B. longum*. Each inoculum (2 μL) was spotted onto the *C. difficile* lawns and incubated in an anaerobic chamber for 48 h. “R” width of the inhibition zone was calculated using the formula $\text{R}=\frac{\left(d\,I\,n\,h\,i\,b\,i\,t-d\,s\,p\,o\,t\right)}{2}$ where d Inhibit denotes the diameter of the inhibition zone around the d spot and d spot denotes the diameter of the spot formed by *B. longum*.

### 2.4. Intracellular and extracellular metabolite sample preparation for the metabolomic analysis

For metabolomics sample preparation, *C. difficile* and *B. longum* were cocultured in 75-mm transwells (3419, Corning, NY, USA) for 10 h. The insert of the transwell was inoculated with *B. longum* at 6.5 × 10^8^ CFU in 9 mL RCM, and the bottom chamber was inoculated with *C. difficile* at 6.5 × 10^7^ CFU in 13 mL RCM. Following coculture for 10 h, equal cells of *C. difficile* in the bottom chamber were harvested based on the OD_600_ and centrifuged (4,000 rpm, 4°C, 15 min) to separate the supernatant and cell pellet. The culture supernatant was stored at −80°C for extracellular metabolite extraction and organic acid. Metabolite sample preparation method was performed based on previous study (Yuan et al., 2012). To reduce changes in the metabolome during metabolite sample preparation, cold solvents were used, and samples were manipulated on ice for all extraction procedures. The cell pellet was washed twice with ice-cold 0.9% NaCl solution to remove any remaining media on the cells. For the intracellular metabolite extraction, 1 mL −80°C 80% methanol spiked with L-[^13^C_9_, ^15^N]phenylalanine (608017, Sigma-Aldrich, MO, USA) at a concentration of 0.5 μM as an internal standard incubated at −80°C for 4 h. The supernatant was subsequently collected *via* centrifugation (13,800 rpm, 4°C, 3 min). Then, the second and third extractions were performed in the same manner for 30 min to pool the extracted metabolites. The extracted metabolite samples were dried using a centrifugal vacuum concentrator (Modulspin 40, Hanil Science Industrial, Korea) and stored at −80°C until analysis.

For hydrophilic extracellular metabolite extraction, 400 μL methanol with 3.3 μM L-[^13^C_9_, ^15^N]phenylalanine and 800 μL chloroform were added to 400 μL culture supernatant. The mixed sample was vortexed for 3 min and then centrifuged (13,800 rpm, 4°C, 3 min). Then, 650 μL of the upper layer mixture was collected and dried using a centrifugal vacuum concentrator. The dried samples were stored at −80°C until analysis.

### 2.5. LC-MS/MS based targeted metabolomics

The dried intracellular and extracellular metabolite samples were dissolved in 40 and 100 μL high-performance liquid chromatography grade water, respectively, and subjected to LC–MS/MS analysis. 1260 Infinity Binary LC (Agilent, CA, USA) combined with 6420 Triple Quadrupole MS (Agilent, CA, USA) was used for the LC–MS/MS analysis. The prepared metabolite sample (10 μL) was injected into the XBridge^®^ Amide column (186004868, 4.6 × 250 mm, particle size 3.5 μm, Waters, MA, USA). Solvent A comprised water/acetonitrile (95:5) with 20 mM ammonium acetate and 20 mM ammonium hydroxide, while solvent B comprised 100% acetonitrile. The LC gradient was: 0 min, 85% B; 5 min, 42% B; 16 min, 0% B; 24 min 0% B; 25 min, 85% B; 32 min, 85% B. The flow rate was 0.4 mL/min. The capillary temperature was 300°C. The electrospray ionization voltage was 4 kV. Agilent MassHunter Qualitative Analysis (version B.07.00) software was used to extract the MS peak areas. The peak area was normalized using the peak area of the internal standard. Statistical analysis of the normalized peak area data was performed using MetaboAnalyst 5.0, and significance was determined using the false discovery rate (FDR) adjusted *p*-value (Pang et al., 2021). The metabolites with fold-change of ≥ 1.5 and a *p*-value of ≤ 0.05 were defined as quantitatively significant.

### 2.6. Protein sample preparation for the proteomic analysis

Cell pellets were obtained in the same way as described above. The obtained cell pellets were resuspended in 1 mL of RIPA lysis and extraction buffer (Thermo Fisher Scientific, MA, USA) containing 0.1% protease inhibitor cocktail (P1860, Sigma-Aldrich, MO, USA) and sonicated using a probe sonicator (Sonics & Materials, CT, USA) for cell lysis. The samples were centrifuged (13,800 rpm, 4°C, 5 min), following which the supernatants were collected. The protein samples were subjected to the filter-aided sample preparation method (Wiśniewski et al., 2009). The protein samples were mixed with 1M dithiothreitol (Sigma-Aldrich, MO, USA) to a final concentration of 50 mM and incubated at 95°C for 5 min. Then, 100 μg protein extract and 200 μL UA buffer (aqueous buffer of 8M urea in 0.1 M Tris/HCl pH 8.5) were mixed and loaded on Microcon-30kDa (MRCF0R030, Merck Millipore, Darmstadt, Germany). The filter unit was centrifuged at 10,000 × *g* for 30 min. Then, 200 μL UA buffer was added to the filter unit and centrifuged at 10,000 × *g* for 15 min, and the process was repeated twice. Then, 100 μL 0.05 M iodoacetamide (Sigma-Aldrich, MO, USA) in UA buffer was added to the filter unit and incubated in the dark for 20 min. The filter unit was centrifuged at 10,000 × *g* for 10 min, following which 100 μL UA buffer was loaded onto the filter and centrifuged at 10,000 × *g* for 15 min. After repeating this process twice, 100 μL 0.05 M Tris/HCl buffer (pH 8.5) was added to the filter unit and centrifuged at 10,000 × *g* for 10 min. This process was repeated twice. The protein sample was subsequently digested by adding 2 μg trypsin and incubating at 37°C for 20 h. To obtain the peptide sample, 250 μL 0.05 M Tris/HCl buffer (pH 8.5) was loaded into the filter unit and centrifuged at 10,000 × *g* for 10 min. Then, the collected peptide samples were desalted using the Pierce Peptide Desalting Spin Columns (89851, Thermo Fisher, MO, USA) according to the manufacturer’s instructions. Finally, the protein samples were dried using a vacuum concentrator and stored at −80°C until analysis.

### 2.7. LC-MS/MS based bottom-up proteomic analysis

Proteomic analysis was performed as described previously (Kwon et al., 2022). The dried peptide samples were reconstituted in solvent A (water/acetonitrile [98:2 v/v] and 0.1% formic acid). For protein analysis, the peptide samples were analyzed using the LC–MS system, which was a combination of Easy-nLC 3000 (Thermo Fisher Scientific, Waltham, MA, USA) coupled with the EASY-spray ion source (Thermo Fisher Scientific, Waltham, MA, USA) on Q-Exactive mass spectrometer (Thermo Fisher Scientific, Waltham, MA, USA). The peptides were separated on the two-column setup using a Acclaim PepMap 100 trap column (100 mm × 2 cm, nanoViper C18, 5 mm, 100 Å, Thermo Scientific, MA, USA) and Acclaim PepMap 100 capillary column (75 mm × 15 cm, nanoViper C18, 3 mm, 100 Å, Thermo Scientific, MA, USA). The peptide sample (2 μg) was first trapped in a trap column and washed with 98% solvent A at a flow rate of 4 μL/min for 6 min. After washing, the sample was separated at a flow rate of 300 nL/min using the capillary column. The LC gradient was run at 2–40% solvent B (100% ACN and 0.1% Formic acid) over 30 min, then from 40 to 95% over 5 min, followed by 95% solvent B (100% ACN and 0.1% Formic acid) for 10 min, and finally 2% solvent B for 20 min. The ion spray voltage was 2,100 eV. Mass data were acquired automatically using Proteome Discoverer 2.5 (Thermo Scientific, USA). Orbitrap analyzer scanned the precursor ions within a mass range of m/z 350–1,800 and a resolution of 70,000 at m/z 200. For collision-induced dissociation, mass spectra were acquired in a data-dependent manner *via* a top 15 method using Q-Exactive. The normalized collision energy (NCE) was 32.

Protein identification and quantification were performed by MaxQuant (version 1.6.17.0) (Cox and Mann, 2008). MS and MS/MS data spectra were queried against the *C. difficile* ATCC 9689 UniProt database (2021.10 released) using the Andromeda search engine. Cysteine carbamidomethylation was set as a fixed modification. Methionine oxidation and N-terminal acetylation were set as variable modifications. Trypsin was used for cleavage, and up to two missed cleavages were allowed. The “match between runs” option was enabled. Proteins and peptides were filtered if they had an FDR of < 1% and quantified *via* label-free quantification (MaxLFQ) (Cox et al., 2014). The statistical analysis of the MaxLFQ data was performed using Perseus software (version 2.0.3.0) (Tyanova et al., 2016), the significance was determined using the adjusted *p*-value. Proteins with a fold-change of ≥ 1.5 and a *p*-value ≤ 0.05 were defined as quantitatively significant.

### 2.8. Organic acid quantification by LC-MS/MS

LC–MS/MS-based organic acid analysis was performed as described previously with some modifications (Kim et al., 2020; Song et al., 2020). The culture supernatant sample was filtered using a polyvinylidene fluoride membrane syringe-driven 0.45-μm pore size filter (SLHVX13NL, Millex-DV, Millipore, MA, USA) to remove cell debris. Next, the filtered sample was transferred to a microtube and diluted 10-fold with water. Then, 50 μl 50% acetonitrile, 40 μl 100 mM N-ethyl-N′-[3-(dimethylamino)propyl]carbodiimide (EDC, 1769, Sigma-Aldrich, MO, USA), 40 μL 100 mM Girard’s reagent T (G900, Sigma-Aldrich, MO, USA), and 10 μL 1 mM sodium [^2^H_7_]butyrate (D5372, CDN ISOTOPES, QC, Canada) were added in 20 μl diluted sample and mixed. The mixture was incubated at 40°C for 1 h, and the samples were diluted 20-fold with 50% acetonitrile. The prepared samples were stored at −20°C until further analysis.

For LC–MS/MS analysis, Acquity UPLC H-Class (Waters, MA, USA) combined with an LTQ XL™ linear ion trap mass spectrometer (Thermo Fisher Scientific, MA, USA) was used. The sample (5 μL) was injected into a Zorbax HILIC plus column (4.6 mm, 100 mm, and 3.5 mm; Agilent, CA, USA). Solvent A comprised water, 20 mM ammonium acetate, and 20 mM acetic acid, while solvent B comprised 100% acetonitrile. The LC gradient was 0 min, 70% B; 1 min, 70% B; 10 min, 30% B; 15 min, 30% B; 15.1 min, 70% B; and 20 min, 70% B. The flow rate was 0.3 mL/min. The NCE was 15–30 eV. For the analysis of organic acids, the previously reported MS/MS detection parameters of 5 GT-labeled short-chain fatty acid and lactate detection parameters (m/z of precursor ion: 204.1; m/z of product ion: 100.1; NCE: 30) were used.

### 2.9. Toxin A quantification using enzyme-linked immunosorbent assay

Toxin A was quantified in culture media using an ELISA kit (TGC-E002-1, tgcBIOMICS, Bingen, Germany). The culture supernatant was obtained *via* centrifugation (13,000 rpm, 3 min, 4°C) of the bottom chamber culture media following coculturing with *B. longum* for 48 h in the transwell. The supernatant was concentrated eight-fold *via* filtration (13,000 rpm, 3 min, 4°C) using a 50 kDa molecular weight cutoff membrane filter (UFC505096, Millipore, MA, USA). Toxin quantification was conducted using the ELISA kit according to the manufacturer’s instructions.

### 2.10. Statistical analysis

Pearson’s correlation coefficient was calculated using IBM SPSS statistics version 27 software (IBM, NY, USA). One-way analysis of variance and post-hoc was conducted using GraphPad Prism version 7 software. The heatmap visualization and PCA of omics datasets were conducted using R statistical programming. Volcano plot was created using VolcaNoseR webtool (Goedhart and Luijsterburg, 2020). For computation of the protein–protein association network and functional enrichment analysis, the STRING database version 11.5 was used (Szklarczyk et al., 2021).

## 3. Results and discussion

### 3.1. Screening of probiotics with growth-inhibitory effects against *C. difficile*

**FIGURE 1:**
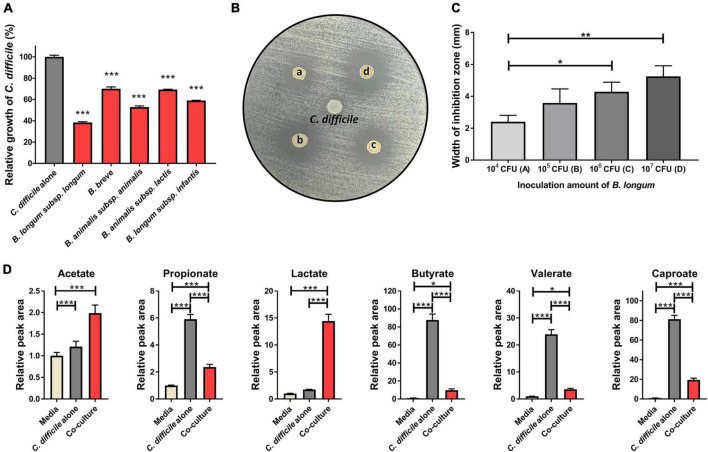
**(A)** Growth inhibition of *C. difficile* due to *Bifidobacterium* strains. Growth inhibition test was performed in triplicate, and the significance was compared with the *C. difficile* alone group. **(B)** Antimicrobial activity of *B. longum* against *C. difficile* was assessed using the spot-on-lawn method. Representative picture showing inoculum density–dependent antimicrobial activity of *B. longum* against *C. difficile*. *C. difficile* was used as a negative control (center spot of agar plate). Circles indicate inhibition zone. (a–d) Indicate the inoculated CFU of *B. longum* (10^4^, 10^5^, 10^6^, and 10^7^ CFU, respectively). **(C)** Width of the inhibition zone (mm) around the *B. longum* spot on the agar plate depends on the inoculated density of *B. longum*. These experiments were performed in triplicate. **(D)** Quantitative comparison of organic acids in culture media (*n* = 4). The significance was calculated *via* one-way analysis of variance and post-hoc (Tukey’s Honestly Significant Difference test). Error bar indicates the standard deviation. The symbols (*), (**), and (***) indicate *p*-value < 0.05, < 0.01, and < 0.001, respectively.

First, to identify the *Bifidobacterium* strains with the highest inhibitory activity against *C. difficile* growth, we cocultured five *Bifidobacterium* strains (*B. longum* subsp. *longum*, *B. longum* subsp. *infantis*, *B. animalis* subsp. *animalis*, *B. animalis* subsp. *lactis*, and *B. breve*) with *C. difficile* in transwells for 10 h. Then, we compared the growth of *C. difficile* with the different strains by measuring optical density at 600 nm (OD_600_) (Figure 1A). Compared with the *C. difficile* monoculture (*C. difficile* alone), all *Bifidobacterium* spp. caused growth inhibition; however, there were significant differences in the extent of growth inhibition among the strains (29.9% for *B. breve* to 61.6% for *B. longum* subsp. *longum*). Interestingly, two bacteria of the same species but different subspecies, *B. longum* subsp. *longum* and *B. longum* subsp. infantis, demonstrated a significant difference in their inhibitory activity (61.6 vs. 40.6%, respectively). In addition, the pH of the culture supernatant decreased by approximately 0.46–0.84 following cocultivation compared with that of the *C. difficile* alone group (Table 1). Several previous studies have reported that organic acids produced by probiotics contribute to *C. difficile* growth inhibition (Naaber et al., 2004; Kolling et al., 2012). This suggests that differences in the production of specific organic acids among *Bifidobacterium* strains may be responsible for the differential inhibitory activity of *Bifidobacterium* spp. against *C. difficile*; however, the underlying mechanisms remain unclear.

**TABLE 1:** pH of the supernatant following coculture of *C. difficile* with *Bifidobacterium* strains (*n* = 3).

Co-cultured *Bifidobacterium* strains	pH	SD	Significance (Compared w/*C. difficile* alone group)
*C. difficile* alone	5.43	0.000	
*B. longum* subsp. *longum* KCTC 3128	4.59	0.017	***
*B. breve* KCTC 3419	4.97	0.012	***
*B. animalis* subsp. *animalis* KCTC 3219	4.93	0.016	***
*B. animalis* subsp. *lactis* KCTC 5854	4.88	0.024	***
*B. longum* subsp. *infantis* KCTC 3249	4.74	0.008	***

The significance was calculated via one-way analysis of variance and *post-hoc* (Tukey’s honestly significant difference test). SD signifies standard deviation. The symbol (***) indicates *p*-value < 0.001.

If *C. difficile* growth inhibition is related to the production of specific organic acid, then *B. longum* subsp. *longum*, the strain with the highest inhibitory activity in the coculture experiment, should exhibit antibacterial activity against *C. difficile*. To confirm this hypothesis, a spot-on-lawn assay was performed to determine the antibacterial potential of *B. longum* against *C. difficile*. We found that *B. longum* formed an inhibition zone, whose width was dependent on the inoculum density of *B. longum* (Figures 1B, C). Next, the organic acids (acetate, propionate, valerate, caproate, butyrate, and lactate) that had accumulated in the cultures following the coculture of *B. longum* and *C. difficile* were quantitatively analyzed using LC–MS/MS to identify the organic acids predominantly produced by *B. longum*. Among the six organic acids, acetate and lactate accumulated significantly more in cocultured with *B. longum* than in the *C. difficile* alone group (Figure 1D). Specifically, lactate and acetate increased by 8.3- and 1.6-fold over the *C. difficile* alone group, respectively (Figure 1D), indicating that lactate was predominantly produced among the organic acids. This result was consistent with those of previous studies involving the same strain of *B. longum* (Yun et al., 2017). Yun et al. (2017) observed an inhibitory effect of *B. longum* on *C. difficile* when the two were cocultured *in vitro*, and this inhibitory effect was dependent on the inoculum density of *B. longum* and the low pH of the culture. The decrease in pH was speculated to be caused by the production of organic acids, including lactic acid, by *B. longum* (Yun et al., 2017). Furthermore, *B. longum* increased the survival rate of *C. difficile*–infected mice and reduced histological damage in the gut. Therefore, lactate was predicted to play a major role in the antibacterial potential of *B. longum* against *C. difficile*.

Other studies have reported on the same species as the *B. longum* strains selected in this study, which have demonstrated growth inhibition of *C. difficile* or alleviation of CDI symptoms. For example, *B. longum* was confirmed to inhibit *C. difficile* growth and reduce its cytotoxicity toward intestinal epithelial cells (HT-29) *in vitro* (Valdés-Varela et al., 2016). Furthermore, *B. longum* reportedly exhibits a growth-inhibitory effect on *C. difficile in vitro* in addition to improving the survival rate of *C. difficile*–infected mice and alleviating intestinal inflammation in a mouse model (Wei et al., 2018). However, despite these studies, the molecular mechanisms underlying the inhibition of *C. difficile* growth and virulence by probiotics remain unclear. Therefore, this study aimed to analyze the physiological changes in *C. difficile* caused by *B. longum* at the molecular level using a multiomics approach to understand these molecular mechanisms.

### 3.2. Proteomic and metabolomic analysis of *C. difficile* cocultured with *B. longum*

**FIGURE 2:**
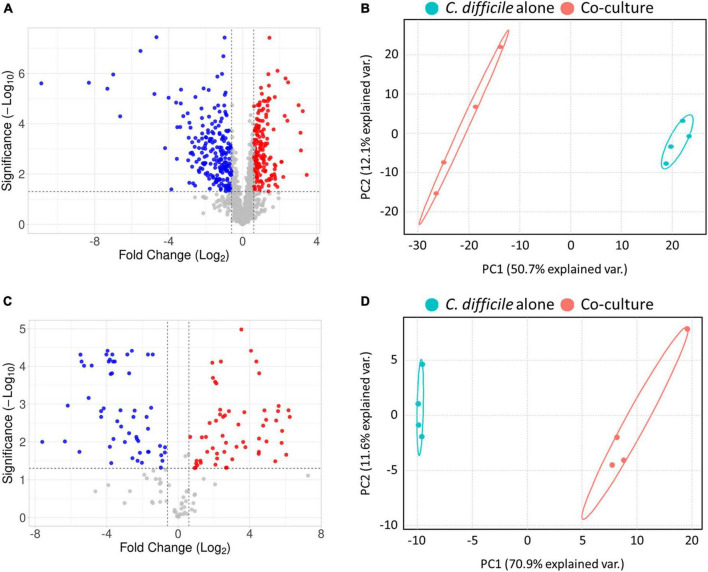
**(A)** Volcano plot of proteomics analysis. **(B)** Principal component analysis (PCA) plot of proteomics analysis. **(C)** Volcano plot of metabolomics analysis. **(D)** PCA plot of metabolomics analysis. In the volcano plot in panel **(A)**, the red and blue colors indicate significantly upregulated and downregulated proteins, and in panel C the red and blue colors indicate metabolites that are at higher and lower levels, respectively, *C. difficile* cocultured with *B. longum* compared to *C. difficile* cultured alone. In the PCA plot, each experimental group was separately clustered. These results indicate that coculture with *B. longum* caused significant proteomic and metabolomic changes in *C. difficile*.

For a comprehensive analysis of the physiological changes in *C. difficile* upon coculture with *B. longum*, we conducted LC–MS/MS-based quantitative proteomics and metabolomics analyses (Supplementary Informations 1–3). Initially, a total of 1,055 proteins were identified following the intracellular proteome analysis, among which the expression levels of 636 proteins remained almost unchanged, those of 234 proteins were downregulated, and those of 185 proteins were upregulated based on the criteria of differentially expressed proteins (DEP, fold-change > 1.5 and *p*-value < 0.05) (Figure 2A). Principal component analysis (PCA) of the proteomics dataset revealed distinct quantitative differences in the proteome of *C. difficile* depending on whether it was cocultured with or without *B. longum* (Figure 2B).

Subsequently, we monitored quantitative changes in intracellular metabolites using an internal multiple reaction monitoring library including 184 metabolites involved in various primary metabolisms. Consequently, we detected 160 intracellular metabolites, with 48 unchanged, 58 upregulated and 54 downregulated metabolites in the *B. longum* coculture group compared with the *C. difficile* alone group (Figure 2C). Moreover, the PCA of the metabolomics dataset revealed that *B. longum* coculture induced quantitative differences in *C. difficile* metabolites, which is consistent with the results of the proteomics data analysis (Figure 2D). Overall, the proteomics and metabolomics analyses demonstrated that *B. longum* coculture induces physiological changes at the molecular level, which are implicated in *C. difficile* growth inhibition.

### 3.3. *B. longum*–produced lactate influences *C. difficile* energy metabolism

**FIGURE 3:**
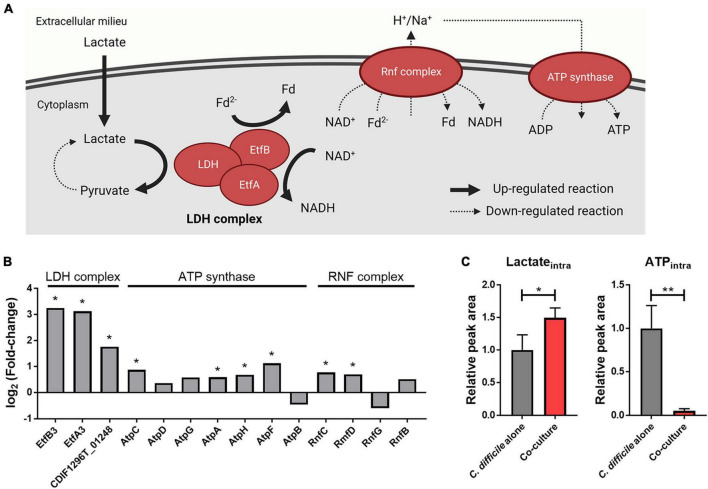
**(A)** Illustration of the effects of lactate reduction on ATP synthesis. This illustration was created using BioRender.com. **(B)** Alterations in intracellular protein levels of the LDH complex, ATP synthase, and Rnf complex. The symbol (*) indicates differentially expressed proteins (DEPs). **(C)** Intracellular protein levels of lactate and ATP. Error bar indicates the standard deviation. The symbols (*) and (**) indicate adjusted *p*-value < 0.05 and < 0.01, respectively.

In our proteomics data, proteins belonging to the lactate dehydrogenase (LDH) complex of *C. difficile* (LDH and electron-transferring flavoproteins) were significantly upregulated upon *B. longum* coculture (Figure 3B). Consistent with this finding, metabolomics data revealed that the intracellular lactate of *C. difficile* increased upon *B. longum* coculture (Figure 3C). Hofmann et al. (2021) reported that L-lactate addition to the culture media induced the expression of an LDH complex–containing operon *via* transcriptome analysis. Therefore, the upregulation of the LDH complex in *C. difficile* cocultured with *B. longum* is attributable to the lactate produced by *B. longum*.

The LDH complex reduces NAD^+^ and metabolizes lactate in the presence of reduced ferredoxin (Fd^2–^) (Weghoff et al., 2015). In addition, previous studies have confirmed that L-lactate added to the medium is metabolized by a LDH complex in *C. difficile* (Hofmann et al., 2021). Therefore, we hypothesized that as lactate produced by *B. longum* increases lactate metabolism in *C. difficile*, changes associated with Fd^2–^ will occur as well in the cellular metabolism of *C. difficile*. Owing to the absence of the classical electron transport chain in *C. difficile*, its alternative, the membrane-spanning Rnf complex, utilizes Fd^2–^ and NADH to generate an ion (i.e., sodium or proton) gradient, which is subsequently utilized by the adenosine triphosphate (ATP) synthase complex for ATP biosynthesis (Neumann-Schaal et al., 2019). In our proteomics and metabolomics analysis, we observed that the Rnf complex and ATP synthase–related proteins either quantitatively remained unchanged or slightly increased in *C. difficile* cocultured with *B. longum* in contrast to the significant decrease in intracellular ATP levels (Figures 3B, C). Thus, the elevated intracellular lactate levels promoted the consumption of Fd^2–^, the Rnf complex substrate during lactate metabolization, by inducing the expression of the LDH complex. Decline in intracellular ATP levels can be attributed to a possible decrease in the intracellular levels of Fd^2–^, which can limit ion gradient generation by the Rnf complex, ultimately leading to reduced ATP production within the cell (Figure 3A). Thus, (1) the large amount of lactate produced by *B. longum* enhances the expression of the LDH complex in *C. difficile*, and (2) the increased lactate oxidation reaction depletes Fd^2–^, thereby inhibiting ion gradient generation by the Rnf complex. (3) This ultimately inhibits ATP biosynthesis *via* the ATP synthase complex, and the resulting lower intracellular ATP level may contribute to *C. difficile* growth inhibition.

### 3.4. *B. longum*–produced proline reduces *C. difficile* toxin production

**FIGURE 4:**
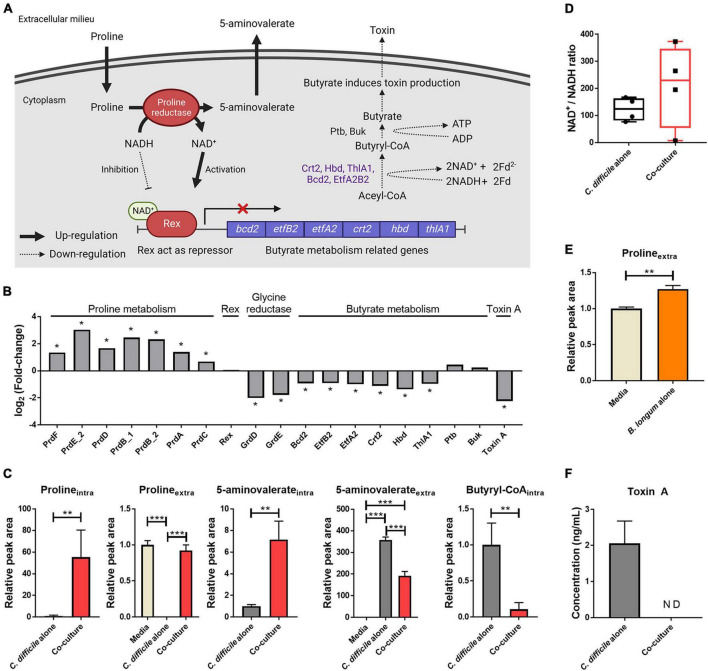
**(A)** Illustration of the effects of proline metabolism upregulation on butyrate metabolism and toxin production. This illustration was created using BioRender.com. **(B)** Alterations in the intracellular protein levels of proline-dependent regulation. The symbol (*) indicates differentially expressed proteins. **(C)** Abundance levels of metabolites belonging to proline reduction and butyrate metabolism. **(D)** Increased levels of the intracellular NAD^+^/NADH ratio in *C. difficile* cocultured with *B. longum*. To measure the NAD^+^/NADH ratio, absolute quantification of NAD^+^ and NADH was performed, and then the NAD^+^ concentration was divided by the NADH concentration of each sample. **(E)** Increased extracellular proline level in *B. longum* monocultured media (*n* = 3). **(F)** Toxin A concentration in culture supernatant after 48 h of cultivation (*n* = 3). Error bar indicates the standard deviation. The symbols (*), (**) and (***) indicate *p*-value < 0.05, < 0.01, and < 0.001, respectively.

The Stickland reaction in the energy metabolism of *C. difficile* produces ATP and NAD^+^ through the oxidation and reduction of amino acids (Neumann-Schaal et al., 2019). *C. difficile* utilizes amino acids, such as proline and glycine, as electron acceptors in the reductive Stickland reaction (Bouillaut et al., 2013). In particular, *C. difficile* converts L-proline to D-proline *via* proline racemase for proline reduction and produces NAD^+^ and 5-aminovalerate using this metabolite (Bouillaut et al., 2013). Initially, our proteomics analysis revealed that proteins involved in proline metabolism, such as proline racemase and D-proline reductase, were significantly upregulated in *C. difficile* upon coculture with *B. longum* (Figure 4B). Intracellular metabolite analysis revealed that the levels of intracellular proline and 5-aminovalerate, the end products of proline metabolism, were significantly elevated in *C. difficile* when co cultured with *B. longum* (Figure 4C). These results demonstrate that the upregulation of proline metabolism in *C. difficile* during coculture with *B. longum* promotes the reductive reaction of proline. However, when cocultured with *B. longum*, the area value of extracellular 5-aminovalerate was 0.54 times lower than that of the *C. difficile* alone group (Figure 4C). In the *C. difficile* alone group, the substrate proline was almost completely depleted (0.002-fold), whereas in the coculture with *B. longum*, the proline levels remained unchanged (Figure 4C). Therefore, the low accumulation of 5-aminovalerate in the culture media despite increased intracellular 5-aminovalerate levels due to elevated proline metabolism may be attributable to a total decreased proline reduction caused by *C. difficile* growth inhibition during coculture with *B. longum*. Notably, despite the considerable production of 5-aminovalerate in the coculture of *C. difficile* and *B. longum*, the level of extracellular proline remained similar to that in the fresh media. Thus, we hypothesized that *C. difficile* consumed the proline produced by *B. longum* while *B. longum* replenished the proline in the culture medium. To confirm this, we performed a quantitative analysis of proline in the culture supernatant of a *B. longum* monoculture and found that the area value of proline was 1.27 times higher than that of the fresh media (Figure 4E). Because proline can induce proline metabolism–related protein expression (Bouillaut et al., 2013), these results indicate that proline produced by *B. longum* is responsible for the upregulation of proline metabolism in *C. difficile*.

In a previous study, proline supplementation to the *C. difficile* media downregulated toxin A expression (Bouillaut et al., 2013). Furthermore, Bouillaut et al. (2019) reported that Rex, an NAD^+^/NADH-responsive regulator, directly binds to DNA and represses the genes involved in alternative NAD^+^-generating pathways (i.e., glycine reductive reaction and butyrate metabolism). They demonstrated that NAD^+^ and NADH act as activators and repressors of the DNA binding activity of Rex, respectively, and that NAD^+^ regeneration by D-proline reductase represses these alternative pathways (Bouillaut et al., 2019). Consistent with the results of these previous studies, the changes in *C. difficile* physiology due to D-proline reductase upregulation were observed in this study (Figure 4). First, an intracellular metabolomic analysis revealed an increased NAD^+^/NADH ratio in *C. difficile* cocultured with *B. longum* (Figure 4D). These results indicate that in *C. difficile* cocultured with *B. longum*, proline metabolism upregulation causes NAD^+^-regeneration, in turn activating Rex. Notably, while the NAD^+^/NADH ratio increased, both NAD^+^ and NADH were significantly reduced in *C. difficile* cocultured with *B. longum* by 14.8- and 29.1-fold, respectively. The NAD^+^ and NADH redox pair is involved as a cofactor in various redox metabolisms, and therefore, the reduction of this metabolite pair in *C. difficile* cocultured with *B. longum* may adversely affect *C. difficile* metabolism and contribute to its growth inhibition. In other words, the total amount of NAD^+^/NADH pair is reduced in *C. difficile* due to the presence of *B. longum*; however, the relative NAD^+^/NADH ratio is increased through the upregulation of NAD^+^ regeneration *via* proline reduction, which activates Rex and inhibits butyrate metabolism.

Second, proteins involved in glycine reductase and butyrate metabolism were downregulated (Figure 4B). Because Rex acts as a repressor that downregulates the expression of these proteins, these results indicate that Rex-dependent regulation occurs due to an increase in the NAD^+^/NADH ratio. Among these downregulated pathways, butyrate metabolism generates energy in *C. difficile* (Figure 4A). As butyrate metabolism generates Fd^2–^ and produces ATP through substrate-level phosphorylation, the downregulation of butyrate metabolism contributes to intracellular ATP level reduction due to decreased ATP production *via* the Rnf complex and ATP synthase and *via* substrate-level phosphorylation. The downregulation of butyrate metabolism was also confirmed by the aforementioned organic acid quantitative analysis (Figure 1C). The production of butyrate decreased to 9.5-fold in the coculture compared with that in the *C. difficile* alone group and decreased to 3.4-fold when the area value was normalized by OD to account for the growth inhibition of *C. difficile*. This result reconfirms the downregulation of butyrate metabolism in *C. difficile*.

Finally, proteomics analysis revealed the downregulation of *C. difficile* toxin A (Figure 4B). Because butyrate acts as a stimulator for toxin production in *C. difficile*, the observed reduction in butyrate production due to the downregulation of its metabolism may contribute to the reduced expression of *tcdA* in *C. difficile* (Karlsson et al., 2000). In addition, we aimed to verify whether the accumulation of toxin A in the culture media was reduced in presence of *B. longum*. Following coculture with *B. longum*, toxin A was quantified *via* enzyme-linked immunosorbent assay (ELISA), and the results revealed that ∼2.1 ng/mL toxin A accumulated in the *C. difficile* alone group, whereas no toxin A was detected in the coculture with *B. longum* (Figure 4F). Thus, proline produced by *B. longum* induces inhibition of toxin production in *C. difficile*, ultimately contributing to the growth and virulence inhibition of *C. difficile*.

### 3.5. *B. longum*–induced proteinaceous stress in *C. difficile*

**FIGURE 5:**
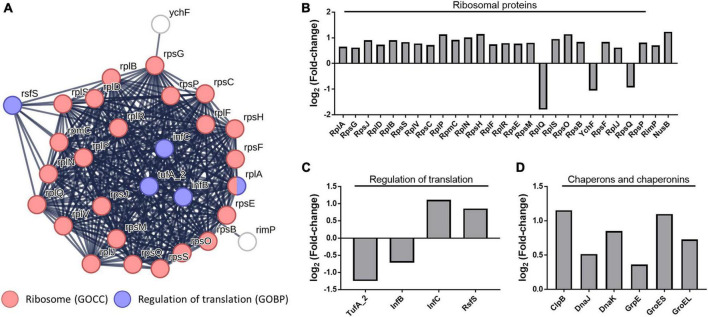
**(A)** Protein–protein interaction network of the gene ontology (GO) terms “ribosome” and “regulation of translation.” GOCC indicates gene ontology cellular Component and GOBP indicates the gene ontology biological process. Alteration of intracellular protein levels related to **(B)** ribosome, **(C)** regulation of translation, **(D)** molecular chaperones, and chaperonins.

As a result of the functional enrichment analysis of DEPs using STRING, the terms “ribosomal protein” and “Regulation of translation” were enriched among the gene ontology (GO) (Figure 5A). In *C. difficile* cocultured with *B. longum*, the ribosomal proteins, ribosome maturation factor (RimP), and transcription antitermination protein (NusB) were upregulated (Figure 5B). RimP is a protein required for the maturation of the 30S ribosomal subunit, and NusB is required for the transcription of ribosomal RNA (rRNA) and functions as an antiterminator in rRNA operons. Together, these results consistently indicate that ribosomal proteins are upregulated in *C. difficile* cocultured with *B. longum*.

Conversely, changes in the abundance of proteins involved in the regulation of translation indicate protein synthesis inhibition. In our proteomics data, the translation initiation factor IF-2 (InfB) and elongation factor Tu (TufA_2), proteins involved in translation regulation, were downregulated, whereas the translation initiation factor IF-3 (InfC) and ribosomal silencing factor (RsfS) were upregulated (Figure 5C) InfB, a GTPase, promotes the binding of N-formylmethionyl-tRNA to the 30S ribosomal subunit, contributing to the formation of the initiation complex (IC), and catalyzes the hydrolysis of GTP to form the 70S ribosome by joining the 50S and 30S IC (Milon et al., 2010). The InfC protein performs two functions: it constitutes the IC and plays a role in dissociating the 70S ribosome into its constituent 30S and 50S ribosomal subunits (Petrelli et al., 2001). TufA_2 is a G protein that participates in translation elongation by catalyzing GTP hydrolysis, which facilitates the binding of aminoacyl-tRNA to the A-site of the ribosome (Agirrezabala and Frank, 2009). Therefore, InfB and TufA_2 downregulation decreased IC formation and impedes translation elongation, whereas InfC upregulation decreases 70S ribosomal subunit formation. This suggests that translation inhibition decreases protein synthesis in *C. difficile* cocultured with *B. longum*. Moreover, RsfS inhibits 70S ribosomal subunit formation in the ribosome (Häuser et al., 2012), indicating that RsfS upregulation inhibits protein synthesis.

Molecular chaperones (ClpB, DnaJ, DnaK, and GrpE) and chaperonins (GroES and GroEL) were observed to be upregulated in *C. difficile* cocultured with *B. longum* (Figure 5D). Chaperons and chaperonins are highly expressed in stressful environments, such as high temperatures, where denatured and aggregated proteins are formed (Kedzierska and Matuszewska, 2001). The Heat shock protein 70 (Hsp70) system proteins (i.e., DnaJ and DnaK) and GrpE, play a role in cooperating with ClpB to refold denatured protein aggregates, thereby ensuring protein quality control and promoting cell survival under stressed conditions (Kedzierska and Matuszewska, 2001). The GroES–GroEL chaperonin system facilitates protein folding and prevents aggregation by creating a nanocage environment favorable for protein folding (Hayer-Hartl et al., 2016). The upregulation of these proteins indicates that *B. longum* induces proteinaceous stress in *C. difficile*. Thus, proteinaceous stress induction causes the differential regulation of ribosomal proteins, translational regulators, and molecular chaperones and chaperonins.

### 3.6. *B. longum* altered the intracellular level of nucleoside phosphates in *C. difficile*

**FIGURE 6:**
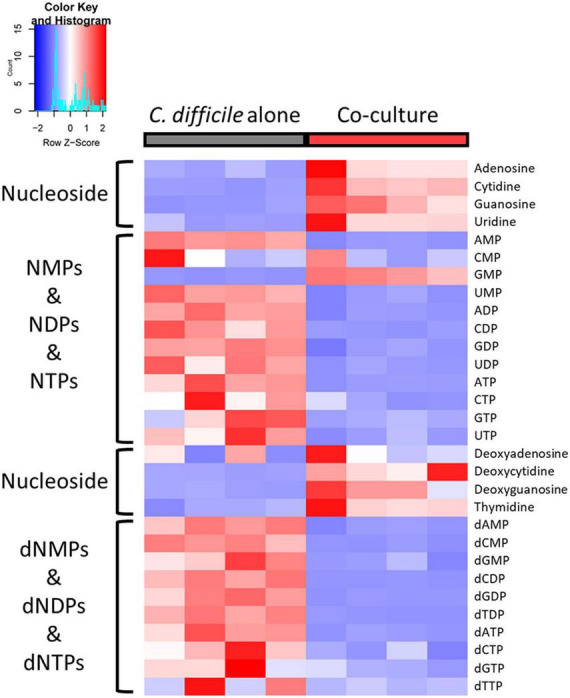
Heatmap of nucleosides and nucleoside phosphates. The red and blue colors indicate relatively high and low abundances, respectively.

Deoxyribonucleoside triphosphate (dNTP) and ribonucleoside triphosphate (NTP), which constitute nucleoside triphosphates, are the building blocks of DNA and RNA, respectively. In our metabolomic analysis, nucleoside phosphates [ribonucleoside monophosphate (NMP), ribonucleoside diphosphate (NDP), deoxyribonucleoside monophosphate (dNMP), and deoxyribonucleoside diphosphate (dNDP)], including all NTPs and dNTPs except GMP, were considerably decreased in *C. difficile* cocultured with *B. longum* (Figure 6). Conversely, nucleosides were increased in *C. difficile* cocultured with *B. longum* (Figure 6). These results suggest that *C. difficile* growth can be inhibited by depleting NTPs and dNTPs, which are directly required for DNA and RNA syntheses. Furthermore, as nucleoside phosphate synthesis requires sequential phosphorylation with ATP, it is reasonable to speculate that nucleoside accumulation and nucleoside phosphate reduction occurred due to low cellular ATP levels. Moreover, decreased GTP levels are another factor that contribute to translation inhibition in *C. difficile*, as GTP is used to activate the aforementioned proteins, including InfB and TufA_2. Therefore, NTP and dNTP depletion in *C. difficile* due to *B. longum* suppresses *C. difficile* proliferation by inhibiting DNA replication, transcription, and translation.

## 4. Conclusion

This study aimed to uncover the molecular mechanisms underlying growth inhibition and reduced toxin production in *C. difficile* due to *B. longum via* the screening of *C. difficile*–inhibiting *Bifidobacterium* spp. and a multiomics approach combining proteomics and metabolomics. First, *B. longum* subsp. *longum*, which exhibited the highest growth inhibition, was screened *via* coculture of *C. difficile* with five *Bifidobacterium* spp. Further, the antimicrobial activity and high lactate production of *B. longum* were confirmed *via* spot-on-lawn assay and LC–MS/MS-based organic acid quantification. Subsequently, we identified numerous proteins and metabolites of *C. difficile* that were significantly altered upon coculture with *B. longum* using quantitative LC–MS/MS-based multiomics analysis. Among these, we proposed the probable mechanisms of the upregulation of LDH and proline metabolism by *B. longum* metabolites, namely lactate and proline, which also contribute to the decreased intracellular ATP levels and toxin production, respectively. Furthermore, proteomics analysis revealed increased ribosomal protein levels, decreased translation-related protein levels, and increased molecular chaperone and chaperonin levels, indicating that *B. longum* induces proteinaceous stress in *C. difficile*. The metabolomics analysis helped us to deduce the mechanism by which decreased nucleoside phosphate levels, including those of (d)NTPs, due to decreased nucleoside phosphorylation inhibits DNA replication, transcription, and translation, leading to the *C. difficile* growth inhibition. Thus, we unveiled the mechanisms of growth and toxin production inhibition in *C. difficile* due to the probiotic bacterium *B. longum via* a multiomics approach. We look forward to identifying the most critical mechanisms of *C. difficile* growth and virulence inhibition through further in-depth studies on such mechanisms and hope to contribute to the development of live biotherapeutic products based on molecular mechanisms for CDI treatment.

## Data availability statement

The data presented in the study are deposited in the ProteomeXchange Consortium via the PRIDE partner repository (Perez-Riverol et al., 2022), accession number PXD040738. All proteomic and metabolomic datasets used in this paper are available in the Supplementary material.

## Author contributions

S-HJ: Conceptualization, Formal analysis, Investigation, Methodology, Project administration, Validation, Visualization, Writing – original draft, Writing – review and editing. H-JJ: Conceptualization, Formal analysis, Investigation, Methodology, Project administration, Validation, Visualization, Writing – original draft. W-SS: Writing – review and editing. J-SL: Writing – review and editing. J-EK; Writing – review and editing. J-HP: Writing – review and editing. Y-RK: Writing – review and editing. M-GK: Writing – review and editing. J-HB: Writing – review and editing. S-YK: Writing – review and editing. J-SK: Writing – review and editing. Y-HY: Writing – review and editing. Y-GK: Conceptualization, Funding acquisition, Project administration, Supervision, Writing – review and editing.
